# Preoperative Frailty Assessed by the Record-Based Multidimensional Prognostic Index Predicts 90-Day Days Alive and out of Hospital Following Radical Cystectomy for Bladder Cancer: A Retrospective Cohort Study

**DOI:** 10.3390/jcm15114057

**Published:** 2026-05-24

**Authors:** Katharina Skovhus, Peter Kristensen, Danny Bech Sindberg, Marianne Ørum, Bente Thoft Jensen, Merete Gregersen, Pernille Skjold Kingo

**Affiliations:** 1 Department of Urology, Aarhus University Hospital, 8200 Aarhus, Denmark; benjense@skejby.rm.dk (B.T.J.); pernking@rm.dk (P.S.K.); 2 Department of Geriatric Medicine, Aarhus University Hospital, 8200 Aarhus, Denmark; marioeru@rm.dk (M.Ø.); meregreg@rm.dk (M.G.); 3 Department of Geriatric Medicine, Regional Hospital Randers, 8930 Randers, Denmark; petkre@rm.dk (P.K.); dabech@rm.dk (D.B.S.); 4 Department of Clinical Medicine, Aarhus University, 8200 Aarhus, Denmark

**Keywords:** days alive and out of hospital, frailty, multidimensional prognostic index, muscle-invasive bladder cancer, radical cystectomy, postoperative recovery

## Abstract

**Background/Objectives**: Radical cystectomy (RC) for muscle-invasive bladder cancer (MIBC) is associated with high morbidity. Frailty is an important determinant of surgical outcomes; however, its association with the composite outcome Days Alive and Out of Hospital (DAOH) has not been examined following RC. We assessed the impact of preoperative frailty on 90-day DAOH in older patients undergoing RC for MIBC. **Methods**: We conducted a retrospective cohort study including 408 consecutive patients aged ≥65 years undergoing RC at a tertiary referral center between 2018 and 2023. Frailty was assessed using the record-based Multidimensional Prognostic Index (r-MPI), classifying patients as non-frail (MPI1), moderately frail (MPI2), or severely frail (MPI3). The primary outcome was 90-day DAOH; secondary outcomes included length of stay (LOS), postoperative complications, delirium, and mortality. DAOH was dichotomized at the cohort median. Associations with low DAOH were analyzed using modified Poisson regression with robust variance estimation. **Results**: Median 90-day DAOH decreased progressively with increasing frailty: MPI1: 81 days (IQR 76–83), MPI2: 73 days (IQR 62–80), MPI3: 67 days (IQR 52–76); *p* < 0.01. In multivariable analysis, frailty was independently associated with low DAOH (MPI2: RR 2.46, 95% CI 1.94–3.11; MPI3: RR 3.37, 95% CI 2.55–4.46), whereas age and comorbidity were not. Increasing frailty was consistently linked to worse postoperative outcomes, including longer LOS, higher complication burden and severity, and more frequent delirium. Ninety-day postoperative complication-related mortality increased markedly with frailty (MPI1: 1.6%, MPI2: 11.9%, MPI3: 12.1%; *p* < 0.01). **Conclusions**: Preoperative frailty is a strong independent predictor of low 90-day DAOH and adverse postoperative outcomes following RC in older patients.

## 1. Introduction

Radical cystectomy (RC) remains the standard curative treatment for muscle-invasive bladder cancer (MIBC) but is associated with substantial perioperative morbidity and mortality. Postoperative complications and prolonged hospitalization are common, particularly in older adults who frequently present with multimorbidity and age-related vulnerability [1]. As the incidence of MIBC increases with age, the proportion of potentially frail patients considered for RC is growing, emphasizing the need for accurate preoperative risk stratification and clinically meaningful outcome measures [2].

Frailty reflects diminished physiological reserve and reduced resilience to perioperative stress, and it is a well-established predictor of adverse surgical outcomes [3,4]. In RC, frailty has been associated with higher complication rates, prolonged hospitalization, functional decline, and increased mortality [1]. However, frailty is not routinely incorporated into preoperative assessment pathways for RC, and evidence linking frailty to global recovery metrics remains limited.

Days Alive and Out of Hospital (DAOH) has emerged as a validated, patient-centered composite outcome. By capturing both early mortality and the cumulative burden of morbidity and hospitalization, DAOH provides a global and comprehensive estimate of the quality and efficiency of postoperative recovery and functional restoration [5,6]. It may be particularly relevant in frail populations where multiple adverse events frequently co-occur and align closely with functional outcomes important to older adults [7]. While frailty has been associated with reduced DAOH in other surgical contexts, no studies have examined the relationship between frailty and DAOH following RC [6,8].

We aimed to investigate the association between preoperative frailty, assessed using the record-based Multidimensional Prognostic Index (r-MPI), and 90-day DAOH in older adults undergoing RC for MIBC or high-risk non-muscle invasive bladder cancer (NMIBC). Secondary endpoints included length of stay (LOS), postoperative complications, delirium, readmissions, and mortality.

## 2. Materials and Methods

### 2.1. Setting and Patients/Population

This retrospective observational cohort study included all consecutive patients aged ≥65 years undergoing RC with an ileal conduit diversion and extended lymph node dissection to the aortic bifurcation for MIBC or high-risk NMIBC at Aarhus University Hospital, Denmark, from January 2018 to May 2023.

### 2.2. Frailty Assessment

Frailty was assessed using the r-MPI, a multicomponent, retrospective frailty tool derived from the original Comprehensive Geriatric Assessment (CGA)-based bedside MPI [9]. The r-MPI enables frailty assessment from routinely documented clinical data [10] and comprises the following domains: Co-habitation status, Polypharmacy, Functional Recovery Score—Activities of Daily Living and Instrumental Activities of Daily Living [11], Cognition [12], Pressure sore risk [13], Comorbidity [14], and Nutrition [15]. The r-MPI was calculated using established methodology [16]; however, given the highly selected surgical cohort, MPI cut-off values were adjusted to improve discrimination between frailty categories (MPI1: 0–0.25; MPI2: 0.26–0.50; MPI3: 0.51–1.00).

### 2.3. Data Collection

Data were retrospectively collected between 1 December 2022 and 31 December 2024. Clinical information was manually extracted from patients’ electronic medical records from six months prior to surgery through the end of follow-up (date of data extraction). Variables included demographics, Performance Status (PS) (0, 1, ≥2), and American Society of Anesthesiologists score (ASA)), tumor characteristics, laboratory values, surgical duration and technique (open/robotic), perioperative bleeding. Frailty was assessed by three experienced geriatric medicine registrars trained in the r-MPI rating procedure. Information on postoperative outcomes, date of discharge, readmissions, complications, and deaths were collected and graded by a single investigator to ensure consistency.

### 2.4. Outcomes

Our primary outcome was 90-day DAOH, calculated as 90 days minus all inpatient days (index admission and readmissions), and further subtracting days lost due to early mortality. Outpatient visits (<24 h) were excluded.

Secondary outcomes included LOS and 90-day postoperative complications graded according to the Clavien–Dindo classification (grades I–V) (CD 1–5) [17]. The cumulative complication burden was quantified using the Comprehensive Complication Index (CCI; range of 0 (none)–100 (death)) [18]. Cognitive complications were classified as documented delirium or “mild confusion,” defined as clinically recorded cognitive deterioration not fulfilling diagnostic criteria for delirium. Mortality outcomes included 90- and 180-day mortality rates, long-term overall survival, and cause-specific mortality attributed to postoperative complications or cancer recurrence.

### 2.5. Statistical Analysis

A priori sample size calculation estimated that 405 patients were required to detect an association between frailty and low 90-day DAOH with 80% power, a two-sided α of 0.05, assuming a 2:1 ratio of non-frail to frail patients, a 40% risk of low DAOH among non-frail patients, and an expected odds ratio of 1.81 associated with frailty. Given the right-skewed distribution of DAOH, the outcome was dichotomized at the cohort median (79 days), defining low DAOH as <79 days. Associations with low DAOH were analyzed using modified Poisson regression with robust variance estimation to obtain risk ratios (RR). Univariable analyses were performed for all candidate predictors. Multivariable models included a priori selected, clinically relevant covariates: MPI category, age, gender, Body Mass Index (BMI), Charlson comorbidity score (CCS grade 0, 1, 2, ≥3), preoperative anemia status (normal/mild, moderate, severe; according to gender-specific WHO thresholds [19]), neoadjuvant chemotherapy (NAC), surgical duration, surgical technique, perioperative bleeding, and tumor stage (T-stage (T1–T4)). Multicollinearity was assessed using generalized variance inflation factors.

Continuous variables are presented as mean (SD) or median (IQR), and categorical variables as counts and percentages. Group comparisons across MPI categories used the Kruskal–Wallis test and Pearson’s chi-squared or Fisher’s exact test, as appropriate.

Overall survival was analyzed using Kaplan–Meier estimates and compared with log-rank tests. Multivariable Cox proportional hazards models were used to estimate adjusted hazard ratios (HR), with proportional hazards assumptions verified by Schoenfeld residuals. Cause-specific mortality was analyzed using cumulative incidence functions with competing risks, and Fine–Gray sub-distribution hazard models were applied for multivariable analyses. A two-sided *p*-value < 0.05 was considered statistically significant. All analyses were performed using R version 4.5.2.

### 2.6. Ethics

The study was registered as a quality improvement project. The protocol was approved by the regional institutional review board at Aarhus University Hospital (Journal Number: 1-49-72-4-18), as required by Danish law.

## 3. Results

### 3.1. Study Population and Baseline Characteristics

Between January 2018 and May 2023, 408 consecutive patients aged ≥65 years underwent RC for MIBC at Aarhus University Hospital and were included in this study. Mean age was 74.6 ± 5.4 years, and 313 patients (77%) were male. Based on r-MPI assessment, 257 patients (63%) were classified as non-frail (MPI1), 118 (29%) as moderately frail (MPI2), and 33 (8%) as severely frail (MPI3). Baseline characteristics across frailty categories are presented in Table 1.

### 3.2. Primary Outcome: 90-Days DAOH

The median 90-day DAOH was 79 days (IQR 71–83). DAOH decreased progressively with increasing frailty severity: MPI1: 81 days (IQR 76–83), MPI2: 73 days (IQR 62–80), MPI3: 67 days (IQR 52–76); *p* < 0.01 (Table 2). The proportion of patients experiencing low DAOH increased significantly across frailty categories (MPI1: 38%; MPI2: 47%; MPI3: 58%; *p* = 0.04). In parallel, median LOS increased with frailty severity.

In univariable analyses (Table 3) increasing frailty was associated with low DAOH (MPI2: RR 2.18, 95% CI 1.75–2.71; MPI3: RR 2.69, 95% CI 2.14–3.39). T4 tumor stage, PS ≥ 2, and abnormal BMI were also associated, while no other covariates reached statistical significance.

In multivariable analyses, frailty remained the strongest independent predictor of reduced DAOH (MPI2: RR 2.46, 95% CI 1.94–3.11; MPI3: RR 3.37, 95% CI 2.55–4.46), with low BMI as the only additional retained covariate. All adjusted GVIF^(1/(2·Df)) values were below 1.32, indicating no concern for multicollinearity among model covariates.

### 3.3. Postoperative Complications

Overall 358 patients (88%) experienced ≥1 postoperative complication within 90 days (Table 2). A total of 1395 postoperative complications were recorded, with a median of three complications per patient (IQR 2–6). Most complications were mild to moderate, with CD2 accounting for 47% of cases. Marked differences were observed across frailty groups.

Delirium occurred in 51 patients (13%), with a stepwise increase across frailty categories (MPI1: 4.7%, MPI2: 21%, MPI3: 42%; *p* < 0.01). Including mild confusion, cognitive complications affected 12% of non-frail patients, 35% of moderately frail, and 51% of severely frail patients.

### 3.4. Readmissions

Within 90 days, 158 patients (39%) required at least one unplanned readmission: 33% of MPI1, 47% of MPI2, and 48% of MPI3 (*p* = 0.02). Multiple readmissions (≥2 readmissions within 90 days) occurred in 54 patients (13%), without significant differences between frailty groups.

### 3.5. Overall Survival

**Figure 1:**
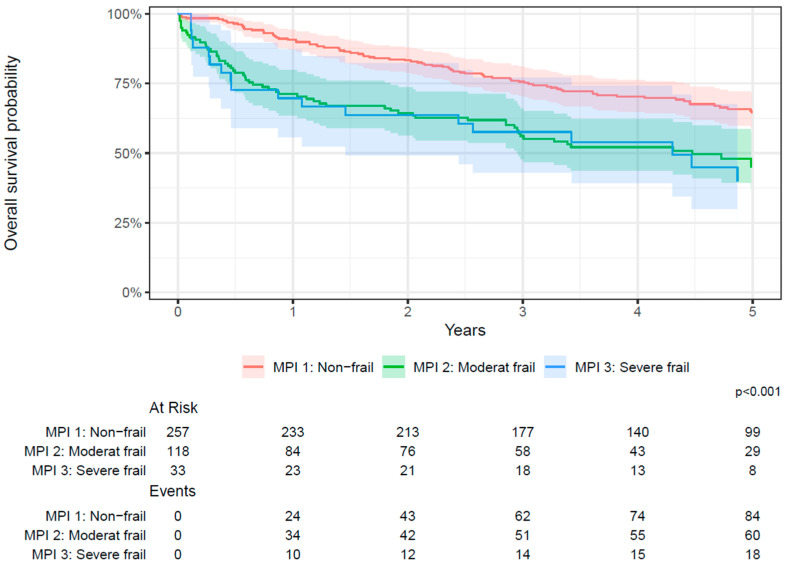
Kaplan–Meier 5-year overall survival curves stratified by frailty/MPI categories.

Overall survival declined progressively with increasing frailty (log-rank *p* < 0.01). Survival curves diverged early and remained separated throughout follow-up. This gradient was evident at 90 days, persisted at 1 year (MPI1: 90.7%; MPI2: 71.2%; MPI3: 69.7%), and remained at 5 years (64.4%, 45.0%, and 40.0%, respectively). See Figure 1.

In univariable Cox analyses, MPI2 and MPI3 were associated with higher mortality compared to MPI1 (MPI2: HR 2.07, 95% CI 1.51–2.82; MPI3: HR 2.21, 95% CI 1.36–3.58) After multivariable adjustment, frailty remained an independent predictor of overall mortality (MPI2: HR 1.81, 95% CI 1.30–2.50; MPI3: HR 1.92, 95% CI 1.16–3.15) (Table 4).

### 3.6. Postoperative Complication-Related Death

Within 90 days after RC, 22 patients (5.4%) died. Mortality rates increased significantly with frailty: MPI1: 1.6%; MPI2: 11.9%; MPI3: 12.1% (*p* < 0.01). Eighteen deaths were attributed to postoperative complications, and four to bladder cancer recurrence. The 90-day cumulative incidence of postoperative complication-related death was 1.6% in MPI1, 8.5% in MPI2, and 12.1% in MPI3 (*p* < 0.01) (Table S1). Deaths among severely frail patients occurred predominantly in days 30–90, whereas non-frail patients died earlier (Figure S1).

At 180 days, complication-related mortality further diverged (MPI1: 1.6%; MPI2: 9.3%; MPI3: 21.2%), with no additional events thereafter. In adjusted Fine–Gray models, both moderate and severe frailty were strongly associated with postoperative mortality (MPI2: HR 6.45, 95% CI 1.99–20.9; MPI3: HR 13.9, 95% CI 3.76–51.6) (Table 4).

### 3.7. Cancer-Specific Mortality

Cancer-specific mortality remained low during the first postoperative year across all frailty groups (MPI1: 7.4%; MPI2: 13.6%; MPI3: 6.1%) (Supplementary Materials). At five years, the estimated cumulative cancer-specific mortality was 23.5% in MPI1, 25.0% in MPI2, and 15.2% in MPI3, whereas competing non-cancer mortality increased progressively across frailty categories (18%, 20%, and 45%, respectively). In adjusted Fine–Gray analyses, frailty was not associated with cancer-specific mortality, while T-stage remained the predominant predictor (T3: HR 4.98, 95% CI 2.60–9.56; T4: HR 7.75, 95% CI 3.65–16.5) (Table 4).

## 4. Discussion

In this retrospective cohort of older patients undergoing RC, preoperative frailty assessed by the r-MPI was significantly associated with reduced 90-day DAOH. DAOH declined progressively across frailty categories, demonstrating a clear severity–outcome relationship, that mirrored increasing complication burden, prolonged LOS, higher readmission rates, and elevated early mortality. After adjustment for age, BMI, comorbidity, PS, T-stage, anemia, and treatment-related factors, frailty remained the strongest independent predictor of low DAOH. No significant interaction between frailty status and surgical technique was observed, suggesting that the association between frailty and reduced DAOH was consistent across surgical approaches. Although DAOH in RC cohorts has previously been linked to postoperative complications and readmissions [20], this study is the first to demonstrate that multidimensional frailty is a key driver of reduced DAOH following RC. Similar associations have been reported in other major surgical populations, where frailty independently predicts prolonged hospitalization and reduced DAOH, suggesting that frailty-related vulnerability operates across surgical disciplines [21].

DAOH is increasingly recognized as a robust patient-centered outcome measure in perioperative research, as it integrates hospitalization, readmissions, and early mortality into a single clinically meaningful metric [5,6]. Unlike isolated endpoints such as LOS or 30-day mortality, DAOH captures the overall quality and efficiency of postoperative recovery and correlates closely with impaired functional recovery, reduced quality of life, postoperative complications, and adverse long-term outcomes [6,22]. Consequently, DAOH has been increasingly applied across diverse perioperative settings, including major abdominal, oncological, emergency, and cardiac surgery cohorts, where lower DAOH consistently correlates with greater perioperative vulnerability and prolonged recovery trajectories [23,24,25,26]. Importantly, this aligns closely with patient-centered priorities, as older adults consistently report valuing time spent at home and preserved independence over survival alone [8,27]. In bladder cancer surgery, DAOH has previously been proposed as a relevant measure of cumulative postoperative morbidity following RC [20], while more recent studies have demonstrated associations between multidimensional geriatric frailty and reduced DAOH in older surgical populations undergoing major cardiac surgery [25]. In the present study, the progressive reduction in DAOH across MPI categories suggests that frailty assessment may identify patients at increased risk of prolonged hospital dependency and impaired postoperative recovery after RC outcomes of substantial relevance not only from a patient perspective but also in terms of healthcare resource utilization.

Low DAOH correlates with impaired functional recovery and reduced quality of life [22] and aligns with patient-centered priorities, as older adults consistently report valuing time spent at home and preserved independence over survival alone [8]. The progressive reduction in DAOH across MPI categories suggests that frailty assessment may identify patients at increased risk of prolonged hospital dependency, an outcome of substantial relevance not only from a patient perspective but also in terms of healthcare resource utilization.

The observed reduction in DAOH among frail patients was paralleled by a substantially higher postoperative complication-related mortality. Both moderate and severe frailty were associated with higher sub-distribution hazards of complication-related death, independent of age, T-stage, comorbidity, and surgical factors. Notably, postoperative mortality followed a distinct temporal pattern. In non-frail patients, complication-related deaths were rare and confined to the first 30 days, whereas deaths among severely frail patients occurred later (days 30–180). This delayed mortality pattern supports the concept of frailty as a state of reduced physiological reserve, characterized by prolonged vulnerability following major surgical stress. Rather than succumbing to immediate perioperative events, frail patients may experience failure to recover from complications, persistent inflammation, and progressive functional decline [28]. These findings challenge the adequacy of short-term endpoints for surgical risk evaluation in frail populations and underscore the relevance of extended follow-up.

Importantly, the reduction in DAOH among frail patients was not explained by mortality alone. Frailty was associated with a higher postoperative complication burden, including more frequent and severe complications and a stepwise increase in CCI. Cognitive complications were particularly pronounced, with delirium rates increasing nearly tenfold from non-frail to severely frail patients. Given the established associations between delirium, prolonged hospitalization, functional decline, and mortality, cognitive vulnerability might contribute to reduced DAOH in frail patients [29]. These findings support systematic delirium prevention strategies and perioperative geriatric co-management, in line with recommendations from the Society of International Oncology in Geriatrics (SIOG) and the European Association of Urology (EAU) that advocate routine cognitive assessment in older cancer patients before RC [30,31].

Long-term overall survival declined progressively with increasing frailty, with estimated five-year survival rates of 64%, 45%, and 40% across MPI categories. After adjustment for age, gender, T-stage, and comorbidity, frailty remained an independent predictor of overall mortality, consistent with prior oncological studies using the MPI [32]. Although severely frail patients demonstrated lower cancer-specific mortality, this apparent advantage was offset by a markedly higher competing risk of non-cancer death, indicating that reduced overall survival was primarily driven by postoperative and frailty-related mortality rather than cancer progression.

Several limitations warrant consideration. Frailty was assessed retrospectively, and some domains—particularly cognition—were incompletely documented, introducing some degree of interpretation or potential misclassification. Nonetheless, r-MPI has demonstrated good prognostic validity in previous cohorts and reflects real-world clinical documentation [16]. Furthermore, the consistency of observed severity-outcome relationships across multiple outcomes supports the robustness of our findings.

MPI cut-offs were adjusted to achieve discrimination in this highly selected surgical cohort, which may limit generalizability. However, the predominance of robust and moderately frail patients likely reflects typical preoperative selection for major surgery. The cut-off values were predefined, resulting in a small severely frail subgroup (n = 33), limiting the precision of effect estimates in this group. Ratings were not blinded, since all data were collected from the same record-system, but outcome-grading was conducted by a single trained assessor to minimize bias and inter-rater variability. No patients were lost to follow-up.

DAOH may be influenced by healthcare system factors such as discharge practices and access to post-acute care. Nevertheless, the median DAOH in our cohort was comparable to prior RC studies, supporting the external validity [20].

Perioperative risk stratification in oncological surgery has traditionally relied on age, comorbidity, and PS—standardized measures of functional capacity and operability. However, PS does not distinguish between underlying causes of impairment [33]. In our study, traditional risk markers were not independently associated with low DAOH after multivariable adjustment, reinforcing evidence that frailty captures vulnerability more comprehensively and that chronological age is a poor surrogate for postoperative resilience [24,34]. The predictive strength of the MPI likely reflects its integration of cognitive, nutritional, and psychosocial domains—factors central to postoperative recovery, but inadequately captured by conventional, unidimensional risk markers. Accordingly, SIOG and EAU guidelines emphasize multidimensional frailty assessment to guide treatment decisions, risk stratification, and supportive care [30,31]. Importantly, frailty assessment should inform perioperative optimization—rather than preclude surgery—including prehabilitation, delirium prevention, and enhanced postoperative follow-up. Whether frailty-guided interventions can improve DAOH in RC remains an important target for future research.

## 5. Conclusions

Preoperative frailty, assessed using the r-MPI, is a strong, independent predictor of reduced 90-day DAOH following RC. Frailty captures multidimensional vulnerability beyond traditional risk markers and demonstrates a clear severity–outcome relationship with cumulative morbidity, cognitive complications, and postoperative mortality. Integrating frailty assessment into perioperative care may improve risk stratification, support shared decision-making, and optimize targeted perioperative care in older patients undergoing RC.

## Figures and Tables

**Table 1:** Patient demographic.

Characteristics	Overall n = 408	Non-Frail (MPI1) n = 257	Moderate Frail (MPI2) n = 118	Severe Frail (MPI3) n = 33
**Age,** year, Mean (±SD)	74.6 (±5.4)	74.2 (±5.4)	75.2 (±5.3)	75.9 (±5.6)
**Gender, Male** (n (%))	313 (77%)	197 (77%)	92 (78%)	24 (73%)
**BMI (kg/m^2^),** Mean (±SD)	26.8 (±4.1)	26.6 (±4.1)	26.7 (±4.3)	28.4 (±4.0)
**Living situation,** n (%)				
Cohabitating	301 (74%)	216 (84%)	78 (66%)	7 (21%)
Alone	102 (25%)	41 (16%)	38 (32%)	23 (70%)
Institutionalized	5 (1.2%)	0 (0%)	2 (1.7%)	3 (9.1%)
**Medication,** n (%)				
0–3 drugs	151 (37%)	127 (49%)	23 (19%)	1 (3%)
4–7 drugs	183 (45%)	107 (42%)	66 (56%)	10 (30%)
≥8 drugs	74 (18%)	23 (8,9%)	29 (25%)	22 (67%)
**Performance Status,** n (%)				
0	239 (59%)	183 (71%)	49 (42%)	7 (21%)
1	152 (37%)	70 (27%)	58 (49%)	24 (73%)
≥2	17 (4.2%)	4 (1.6%)	11 (9.3%)	2 (6.1%)
**Charlson Comorbidity Score,** n (%)				
0	109 (27%)	89 (35%)	18 (15%)	2 (6.1%)
1	103 (25%)	65 (25%)	32 (27%)	6 (18%)
2	90 (22%)	56 (22%)	27 (23%)	7 (21%)
≥3	106 (26%)	47 (18%)	41 (35%)	18 (55%)
**ASA score,** n (%)				
1	16 (3.9%)	16 (6.2%)	0 (0%)	0 (0%)
2	214 (52%)	156 (61%)	47 (40%)	11 (33%)
≥3	178 (44%)	85 (33%)	71 (60%)	22 (67%)
**Preoperative NAC,** n (%)	98 (24%)	69 (27%)	23 (19%)	6 (18%)
**Preoperative hemoglobin, mmol/L,** Mean (±SD)	7.83 (±1.17)	7.93 (±1.15)	7.68 (±1.15)	7.58 (±1.27)
**Surgical technique,** n (%)				
Open surgery	160 (39%)	97 (38%)	47 (40%)	16 (48%)
Robotic surgery	248 (61%)	160 (62%)	71 (60%)	17 (52%)
**Surgical duration (min),** Mean (±SD)	331 (±89)	329 (±85)	331 (±99)	348 (±85)
**Perioperative bleeding (mL),** Median (IQR)	400 (150, 900)	300 (150, 800)	400 (150, 1100)	725 (250, 1100)
Tumor stage *, n (%)				
T1 + CIS	119 (29%)	86 (33%)	26 (22%)	7 (21%)
T2	168 (41%)	102 (40%)	53 (45%)	13 (39%)
T3	86 (21%)	53 (21%)	22 (19%)	11 (33%)
T4	35 (8.6%)	16 (6.2%)	17 (14%)	2 (6.1%)

SD = Standard deviation; IQR = Interquartile range; MPI = Multidimensional Prognostic Index; BMI = Body Mass Index; ASA = American Society of Anesthesiologist Physical Status Classification System; NAC = Neoadjuvant chemotherapy; * Tumor stage = highest pathological stage diagnosed after either transurethral resection of the bladder (TURB) or radical cystectomy.

**Table 2:** Ninety-day postoperative outcomes following radical cystectomy stratified by Multidimensional Prognostic Index (MPI) category.

Characteristic	Overall n = 408	MPI1 n = 257	MPI2 n = 118	MPI3 n = 33	*p*-Value
**DAOH 90-days (d), Median (IQR)**	79 (71, 83)	81 (76, 83)	73 (62, 80)	67 (52, 76)	<0.01
**LOS (d), Median (IQR)**	8 (7, 12)	7 (7, 9)	10 (8, 15)	15 (9, 30)	<0.01
Readmission (1) *, n (%)	158 (39%)	86 (33%)	56 (47%)	16 (48%)	0.02
Medical complication	81 (51.2%)	47 (54.7%)	23 (41.1%)	11 (68.8%)	
Surgical complication	67 (42.4%)	34 (39.5%)	28 (50%)	5 (31.3%)	
Other reason	11 (7.0%)	6 (7.0%)	5 (8.9%)	0 (0%)	
Unknown	1 (0.6%)	1 (1.2%)	0 (0%)	0 (0%)	
Readmission (≥2) *, n (%)	54 (13%)	27 (11%)	21 (18%)	6 (18%)	0.09
**Complications (90-d)**					
Total number of complications per patient, median (IQR)	3 (1, 5)	2 (1, 3)	4 (2, 7)	6 (3, 8)	<0.01
Any complication, n (%)	358 (88%)	211 (82%)	115 (97%)	32 (97%)	<0.01
Minor complication (CD1–2), n (%)	332 (81%)	198 (77%)	105 (89%)	29 (88%)	0.01
Major complication (CD ≥ 3a), n (%)	155 (38%)	64 (25%)	70 (59%)	21 (64%)	<0.01
**CCI, Median (IQR)**	34 (21, 50)	26 (21, 37)	47 (36, 63)	60 (44, 70)	<0.01
**Highest CD grade, n (%)**					
None	50 (12%)	46 (18%)	3 (2.5%)	1 (3.0%)	
CD1	12 (2.9%)	12 (4.7%)	0 (0.0%)	0 (0.0%)	
CD2	191 (47%)	135 (53%)	45 (38%)	11 (33%)	
CD3a	72 (18%)	35 (14%)	30 (25%)	7 (21%)	
CD3b	34 (8.3%)	17 (6.6%)	16 (14%)	1 (3.0%)	
CD4a	27 (6.6%)	8 (3.1%)	13 (11%)	6 (18%)	
CD4b	4 (1.0%)	0.0 (0.0%)	1 (0.8%)	3 (9.0%)	
CD5	18 (4.4%)	4 (1.6%)	10 (8.5%)	4 (12%)	
**Cognitive complications, n (%)**					<0.01
Mild confusion	40 (9.8%)	20 (7.8%)	17 (14%)	3 (9.1%)	
Delirium	51 (13%)	12 (4.7%)	25 (21%)	14 (42%)	

Mean (±standard deviation (SD)), Median (Interquartile range (Q1–Q3)), Number (n (%)); DAOH = Days Alive and Out of Hospital; CD = Clavien–Dindo classification; CCI = Comprehensive Complication Index; MPI = Multidimensional Prognostic Index. Group comparisons were performed using the Kruskal–Wallis rank-sum test for continuous variables and Pearson’s χ^2^ test or Fisher’s exact test for categorical variables, as appropriate. * Readmission (1) = First/one readmission. Readmission (≥2) = Two or more readmissions.

**Table 3:** Univariable and multivariable modified Poisson regression analyses presenting risk ratios (RR) with 95% confidence intervals (95% CI) for low days alive DAOH (≤79 days) following radical cystectomy.

Characteristic	Crude RR *	95% CI	Adjusted RR *	95% CI
**Frailty**				
MPI 2: Moderate frailty	2.18	(1.75–2.71)	2.46	(1.94–3.11)
MPI 3: Severe frailty	2.69	(2.14–3.39)	3.37	(2.55–4.46)
**Age**				
70–74 years	0.97	(0.71–1.32)	0.77	(0.57–1.04)
75–79 years	1.03	(0.77–1.37)	0.85	(0.64–1.13)
≥80 years	0.95	(0.68–1.34)	0.82	(0.58–1.16)
**Gender**				
Male	1.04	(0.81–1.33)	1.06	(0.83–1.36)
**Body Mass Index (BMI)**				
Underweight (<18.5)	1.99	(1.08–3.64)	1.98	(1.14–3.45)
Overweight (25–29.9)	1.34	(1.03–1.73)	1.23	(0.95–1.59)
Obese (≥30)	1.34	(0.99–1.81)	1.28	(0.95–1.72)
**Charlson Comorbidity Score**				
1	0.99	(0.74–1.34)	0.81	(0.61–1.07)
2	1.06	(0.79–1.43)	0.92	(0.67–1.26)
≥3	1.09	(0.82–1.45)	0.81	(0.60–1.08)
**Performance Status**				
1	1.11	(0.89–1.38)	0.83	(0.67–1.04)
≥2	1.47	(1.01–2.15)	1.00	(0.70–1.44)
**Anemia status**				
Moderate anemia	0.96	(0.66–1.22)	0.96	(0.76–1.21)
Severe anemia	0.92	(0.69–1.23)	0.75	(0.54–1.03)
**Neoadjuvant chemotherapy**				
Yes	1.01	(0.79–1.29)	1.13	(0.86–1.50)
**Surgical technique**				
Robotic Surgery	1.03	(0.83–1.28)	0.92	(0.66–1.30)
**Surgery duration**				
4–6 h	0.90	(0.66–1.22)	1.03	(0.77–1.37)
≥6 h	0.99	(0.73–1.35)	1.00	(0.74–1.35)
**Perioperative bleeding**				
150–400 mL	0.94	(0.70–1.27)	0.87	(0.64–1.18)
401–900 mL	0.97	(0.72–1.32)	1.03	(0.72–1.47)
>900 mL	0.96	(0.71–1.30)	0.78	(0.51–1.19)
**Tumor stage (T stadium)**				
T2	1.24	(0.96–1.61)	1.11	(0.86–1.44)
T3	0.96	(0.68–1.35)	0.90	(0.65–1.25)
T4	1.46	(1.03–2.06)	1.25	(0.86–1.82)

* References: MPI1: Non-frail; Age: <70 years; Gender: Female; BMI: Normal (18.5–24.9); Charlson Comorbidity Score: 0; Performance Status: 0; Anemia status: Normal/mild anemia; Neoadjuvant chemotherapy: No; Surgical technique: Open surgery; Surgery duration: <4 h; Perioperative bleeding: <150 mL; Tumor stage: T1.

**Table 4:** Crude and adjusted hazard ratios (HRs) for overall survival, postoperative complication-related death, and cancer-specific death according to record-based MPI frailty categories.

	Crude HR	95% CI	*p*-Value	Adjusted HR	95% CI	*p*-Value
Overall survival *						
MPI1, non-frail	-	-	-	-	-	
MPI2, moderate frail	2.07	1.51–2.82	<0.01	1.81	1.30–2.5	<0.001
MPI3, severe frail	2.21	1.36–3.58	<0.01	1.92	1.16–3.15	0.011
Postoperative complication-related death **						
MPI1, non-frail	-	-	-	-	-	
MPI2, moderate frail	6.27	2.0–19.7	0.002	5.49	1.71–17.7	0.004
MPI3, severe frail	14.1	4.13–48.2	<0.001	13.9	3.76–51.6	<0.001
Cancer-specific death ***						
MPI1, non-frail	-	-	-	-	-	
MPI2, moderate frail	1.25	0.82–1.91	0.3	1.14	0.72–1.79	0.6
MPI3, severe frail	0.76	0.34–1.73	0.5	0.64	0.27–1.49	0.3

* CI = Confidence Interval, HR = Hazard Ratio; 95% CI = 95% confidence intervals. * Cox proportional hazards regression with corresponding log-rank tests, adjusted for age, sex, tumor stage, and Charlson Comorbidity Score. Postoperative complication-related mortality and cancer-specific death were analyzed using Fine–Gray competing risk regression models. ** Adjusted for age, body mass index, T stage, Charlson Comorbidity Score, and surgical technique. *** Adjusted for age, T stage, Charlson Comorbidity Score, and neoadjuvant chemotherapy.

## Data Availability

The datasets generated in this study are subject to legal and ethical restrictions and cannot be made publicly available. According to the project approval, individual-level data may only be used within the approved study and cannot be shared without prior authorization Danish Health Authorities.

## Supplementary Materials

The following supporting information can be downloaded at: https://www.mdpi.com/article/10.3390/jcm15114057/s1. Figure S1: One-year cumulative incidence of postoperative complication-related death stratified by frailty (MPI) category; Table S1: Long-term survival outcomes and cumulative incidence of cause-specific mortality stratified by frailty (MPI) category.

## Abbreviations

The following abbreviations are used in this manuscript:

RC	Radical Cystectomy
MIBC	Muscle-invasive Bladder Cancer
NMIBC	Non-muscle Invasive Bladder Cancer
DAOH	Days Alive and Out of Hospital
r-MPI	Record-based Multidimensional Prognostic Index
MPI	Multidimensional Prognostic Index
LOS	Length of Stay
CGA	Comprehensive Geriatric Assessment
PS	Performance Status
ASA	American Society of Anesthesiologists score
CD	Clavien–Dindo
CCI	Comprehensive Complication Index
CCS	Charlson Comorbidity Score
NAC	Neoadjuvant Chemotherapy
HR	Hazard Ratio
RR	Relative Risk
