# Supplementary material for: Preoperative Frailty Assessed by the Record-Based Multidimensional Prognostic Index Predicts 90-Day Days Alive and out of Hospital Following Radical Cystectomy for Bladder Cancer: A Retrospective Cohort Study
Source: J Clin Med. 2026 May 24;15(11):4057. doi: 10.3390/jcm15114057 (PMC13257800; doi:10.3390/jcm15114057)
Supplement: Supplementary file 1 [file jcm-15-04057-s001.zip › Figure S1.pdf]

Figure S1: One-year cumulative incidence of postoperative complication-related death stratified by frailty (MPI) category.

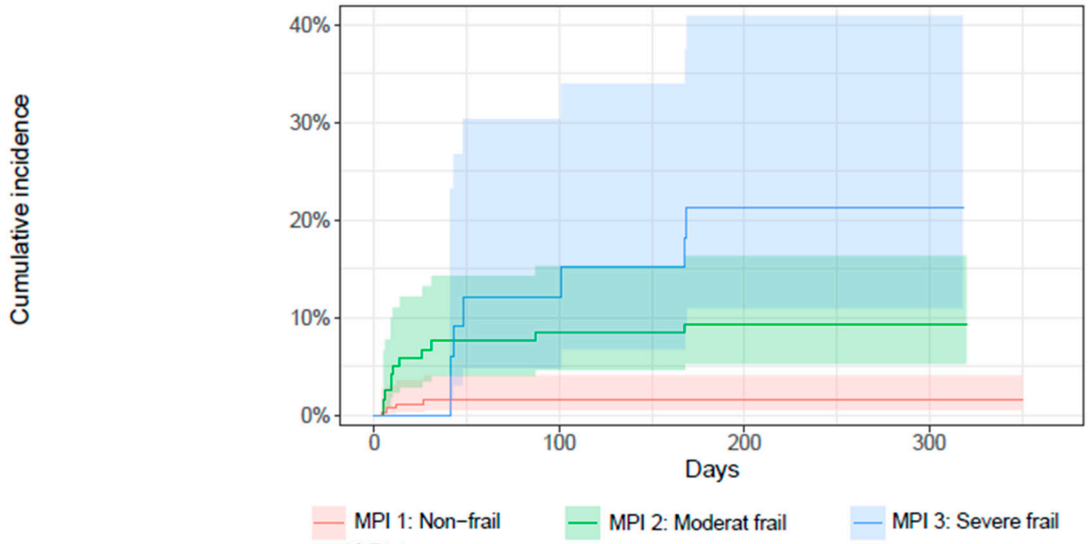

At Risk

MPI 1: Non−frail	257	253	247	239
MPI 2: Moderat frail	118	103	93	86
MPI 3: Severe frail	33	28	24	24

Events

MPI 1: Non−frail	0	4	4	4
MPI 2: Moderat frail	0	10	11	11
MPI 3: Severe frail	0	4	7	7
