# Supplementary material for: Preoperative Frailty Assessed by the Record-Based Multidimensional Prognostic Index Predicts 90-Day Days Alive and out of Hospital Following Radical Cystectomy for Bladder Cancer: A Retrospective Cohort Study
Source: J Clin Med. 2026 May 24;15(11):4057. doi: 10.3390/jcm15114057 (PMC13257800; doi:10.3390/jcm15114057)
Supplement: Supplementary file 1 [file jcm-15-04057-s001.zip › Table S1.pdf]

Tabel S1: Long-term survival outcomes and cumulative incidence of cause-specific mortality stratified by frailty (MPI) category.

	**Overall**<br>n = 408	**MPI 1**<br>n = 257	**MPI 2**<br>n = 118	**MPI 3**<br>n = 33
**Overall survival rates, % (95% CI)***				
**90 days**	94.6 (92.4-96.8)	98.4 (96.9-100)	88.1 (82.5-94.2)	87.9 (77.4-99.8)
**1 year**	83.3 (79.8-87.0)	90.7 (87.2-94.3)	71.2 (63.5-79.8)	69.7 (55.7-87.3)
**2 years**	76.2 (72.2-80.2)	83.3 (78.8-88.0)	64.4 (56.3-73.7)	63.6 (49.2-82.4)
**5 years**	56.9 (51.9-62.3)	64.4 (58.4- 71.0)	45.0 (36.1-56.0)	40.0 (25.0-63.9)
**Cumulative incidence of postoperative complication-related death, % (95% CI)****				
**30 days**	2.9 (1.7-5.1)	1.6 (0.6-4.1)	6.8 (3.5-13.2)	0.0 (0.0-0.0)
**90 days**	4.2 (2.8-6.9)	1.6 (0.6-4.1)	8.5 (4.7-15.3)	12.1 (4.8-30.4)
**180 days**	5.2 (3.6-8.1)	1.6 (0.6-4.1)	9.3 (5.3-16.4)	21.2 (11.0-40.9)
**1 year**	5.2 (3.6-8.1)	1.6 (0.6-4.1)	9.3 (5.3-16.4)	21.2 (11.0-40.9)
**Cumulative incidence of cancer-specific death (%)****				
**1 year**	9.1 (6.7-12.3)	7.4 (4.8-11.4)	13.6 (8.6-21.4)	6.1 (1.6-23.2)
**2 years**	15.4 (12.3-19.4)	14.0 (10.3-19.0)	19.5 (13.5-28.1)	12.1 (4.8-30.4)
**5 years**	22.8(19.5-27.9)	23.5 (18.7-29.6)	25.0 (18.2-34.3)	15.2 (6.8-34.0)

*Overall survival rates were estimated using the Kaplan-Meier method.

**Cumulative incidence functions accounted for competing risks of death from other causes.

MPI 1 = robust patients; MPI 2 = moderately frail patients; MPI 3 = severely frail patients. CI = confidence interval; MPI = Multidimensional Prognostic Index."
